# Anti-CD137 agonist antibody–independent and clinically feasible preparation of tumor-infiltrating lymphocytes from soft tissue sarcoma and osteosarcoma

**DOI:** 10.3389/fimmu.2025.1557006

**Published:** 2025-03-12

**Authors:** Yining Jin, Zhiliang Jia, Xueqing Xia, Nancy B. Gordon, Joseph A. Ludwig, Neeta Somaiah, Shulin Li

**Affiliations:** ^1^ Department of Pediatrics, The University of Texas MD Anderson Cancer Center, Houston, TX, United States; ^2^ Department of Sarcoma Medical Oncology, The University of Texas MD Anderson Cancer Center, Houston, TX, United States

**Keywords:** immunotherapy, tumor-infiltrating lymphocytes, sarcoma, adoptive cell therapy, GMP

## Abstract

**Background:**

Tumor infiltrating lymphocytes (TILs) therapy has been proved for treatment of metastatic melanoma and is under investigation for other types of solid tumors. However, these successes are threatened by discontinued supply of GMP-grade anti-CD137 agonist, a key TIL preparation reagent. Therefore, exploring a GMP-adherent method for expanding endogenous TILs without anti-CD137 agonist is urgent. Toward this end, we aimed to establish an anti-CD137–independent and clinically feasible TIL expansion protocol to prepare TILs from under investigated sarcoma tumors.

**Methods:**

We collected resected tumors from patients and cut tissues into fragments. We used IL-2 and T-cell activator CD3/CD28 without anti-CD137 agonist to expand nonselected TILs in 2-3 weeks, then rapidly expanded them over 2 weeks. Their phenotypes were characterized using flow cytometry. Their antitumor activity was validated *in vitro* using cytotoxic T lymphocyte assays measuring CD107a on the TILs and the viability of tumor cells and *in vivo* using an autologous patient-derived xenograft (PDX) tumor model.

**Results:**

We successfully expanded TILs in > 90% of collected samples. TILs generated preferentially increased CD8+ T cells but suppressed CD4+ T cells. A small portion of TILs were resident memory T cells. The expanded TILs reduced autologous tumor cells by 37.5% within 24 hours. Infusion of TILs in mice bearing autologous PDX tumors strongly inhibited liposarcoma growth. FDA has approved use of this GMP-feasible protocol in our clinical trial (IND 30562).

**Conclusion:**

It is feasible to generate antitumor TILs using CD3/CD28 activator to replace the unavailable anti-CD137 agonist. Our study supports the further development of TIL-based therapy.

## Introduction

In adoptive cell therapy (ACT) with tumor-infiltrating lymphocytes (TILs), a patient’s own tumor-responsive immune cells are isolated from the tumor microenvironment, expanded *in vitro*, genetically engineered to enhance their antitumor efficacy, and infused back into the patient to eliminate cancer. The traditional method of generating TILs starts with a high dose of interleukin (IL)-2 to induce the migration of TILs out of the tumor fragments and their proliferation in a 24-well plate. TILs generated by this method have been reported to be highly effective against melanoma (1, 2). To further improve TIL expansion technology, particularly the proportion of CD8+ TILs, and to enhance antitumor activity, a 3-signal approach, TIL 3.0, was developed (2). This novel culture method included agonistic antibodies against CD3 and its co-stimulatory signal CD137 (urelumab) in combination with IL-2 in a gas-permeable rapid expansion (G-Rex) flask (3). CD137 provides a second signal for T-cell activation and enhances their antitumor response (4). Several clinical trials using TILs generated by TIL 3.0 have been conducted (e.g., NCT00338377, NCT03610490, and NCT02652455).

TILs generated by the TIL 3.0 method are effective against melanoma and other solid tumors, but the anti-CD137 agonist antibody urelumab is no longer available due to its liver toxicity and limited clinical activity when it was used as monotherapy (5). Therefore, we adapted the 3-signal approach to generate TILs using an antibody targeting the T-cell co-stimulatory signal CD28 instead of CD137. Ligation of the co-stimulatory CD28 receptor on T cells, which mimics T-cell stimulation by antigen-presenting cells, provides a vital second signal for T-cell activation and activation of the T-cell receptor (TCR)-CD3 signaling complex. We used CTS Dynabeads CD3/CD28 (Thermo Fisher) to generate pre-rapid expansion phase (REP) TILs. CTS Dynabeads CD3/CD28 are intended for *ex vivo* expansion of human T cells in clinical settings and have been successfully tested in a few clinical trials (6, 7). In addition, once TILs start to proliferate, beads and T cells form aggregates that can be easily observed under the microscope by the brown color imparted by the beads, making the T cells distinguishable from other cells and fragments of tissue.

While current TIL-based ACT clinical trials have been tested mostly in immunogenic or “hot” tumors such as melanoma, non-small cell lung cancer, and cervical cancer, very few studies of the application of TILs in immunologically nonresponsive or “cold” tumors have been reported. To test the feasibility of our method in “cold” tumors, we applied it to generate TILs from freshly resected tissue obtained from sarcoma patients because these samples are accessible in the clinical context.

Soft tissue sarcomas are rare and diverse cancers that emerge from oncogenic transformation of connective tissues and can invade surrounding tissues and organs (8). Liposarcoma emerges in adipocytes, often develops in the extremities and retroperitoneum, and presents unique challenges in diagnosis and treatment (9). The primary treatment for liposarcoma is surgical resection followed by either radiotherapy and/or chemotherapy to kill the remaining cancer cells. However, liposarcoma has high rates of recurrence and metastasis, and the standard treatment approach offers only a limited period of disease control (10). Thus, there is an urgent need for more effective and less toxic liposarcoma treatments that offer better coverage of this tumor’s heterogeneity and long-term disease control. ACT of patients’ naturally occurring T cells, which can target their cancer cells, has emerged as a potential treatment.

In light of the unavailability of the anti-CD137 agonist antibody used to expand autologous TILs, the aims of this study were to establish methods for the expansion of tumor-reactive TILs from freshly resected soft tissue sarcoma in a clinically relevant, GMP-adherent manner and to test the method for other types of sarcomas, such as osteosarcoma. This TIL expansion protocol includes a stage of pre-REP expansion and a subsequent REP expansion. In the pre-REP phase, our protocol uses high-dose IL-2 in combination with CTS Dynabeads CD3/CD28; in the REP procedure, it uses OKT anti-CD3 antibody and irradiated peripheral blood mononuclear cells (PBMCs) as feeder cells. To enable the clinical application of our protocol, we generated “young TILs” without enrichment for tumor reactivity. Our data demonstrated the feasibility of expanding functional TILs from fresh soft tissue sarcoma.

## Materials and methods

### Reagents for pre-REP culture

Pre-REP TILs were cultured in RPMI 1640 (Mod.) 1X with L-glutamine, HEPES (Corning, NY, USA), 6000 IU/mL recombinant human (rh) IL-2, proleukin (Prometheus Laboratories, San Diego, USA), CTS Dynabeads CD3/CD28 (Thermo Fisher Scientific, Waltham, USA), and human serum AB (GeminiBio, West Sacramento, USA). Antibiotic-Antimycotic Solution (Corning, NY, USA) was added in the pre-REP culture medium for the first week.

### Reagents for REP culture

REP culture medium for the first week of expansion consisted of RPMI 1640 mixed at a 1:1 ratio with CTS AIM-V medium (Thermo Fisher Scientific, Waltham, USA), 30 ng/mL OKT3 (anti-CD3) antibody (Miltenyi Biotec, Bergisch Gladbach, Germany), and 3000 IU/mL rhIL-2. Allogeneic PBMCs were obtained from buffy coats from healthy donors (The University of Texas MD Anderson Cancer Center Blood Bank, Houston, USA), irradiated at 100 Gy, and used as feeder cells.

Hank’s Balanced Salt Solution (HBSS) (Corning, NY, USA) was used for rinsing tumor fragments.

### Disposables

We used nontreated T-25 tissue culture flasks (VWR, West Chester, USA), nontreated T-75 tissue culture flasks (VWR, West Chester, USA), nontreated 24-well tissue culture plates (Corning, NY, USA), 60 mm * 15 mm Petri dishes (Thermo Fisher Scientific, Waltham, USA), 96-well cell culture plates, F-bottom clear 96-well plates (Greiner Bio-One, Monroe, USA), and a G-Rex 10M Open System (Wilson Wolf, **St. Paul, USA**).

### Tumor samples

Liposarcoma, osteosarcoma, and neuroblastoma samples were collected from spared surgical tumors under an Institutional Review Board–approved protocol (2020-0465-MOD012) at The University of Texas MD Anderson Cancer Center. Informed and written consent was obtained from all the patients involved in this project.

### Tumor tissue fragment preparation

Immediately after retrieval, fresh tumor specimens were transported to the laboratory in RPMI 1640 medium on ice. The specimens were transferred to a Petri dish, rinsed, and covered with HBSS. Tumors were then cut with scissors into multiple fragments of approximately 1 to 3 mm^3^ each (width × length: 1-1.5 mm × 1-1.5 mm).

### Pre-REP TIL culture generation

At day 0, 5 fragments representative of the whole tumor were placed in one well of a 24-well plate with 1.5 mL of pre-REP TIL culture medium. Several wells were prepared for the same tissue. Three microliters of CD3/CD28 Dynabeads were added into each well. The plate was then incubated at 37°C, 5% CO_2_ for TIL culture.

During the first week of culture (day 5), rhIL-2 was added (9 μL of 10^6^ IU/mL rhIL-2/well). After the first week of culture, each well was monitored for TIL confluence every 2 to 3 days or as needed. TILs were considered confluent when they formed a thick layer of cells that covered 100% of the bottom of the well or when the media had turned yellow. Half of the medium was replaced in wells with confluent cells.

At the third week, if a well again contained confluent cells, TILs in the well were gently mixed by pipetting with a 2 mL serological pipet. An aliquot was taken from the resuspended well. Cells were counted and their viability determined. If the target number of 1 × 10^6^ TILs per well was not achieved, the cells were further cultured. Otherwise, cells were pooled with other wells representing the same tissue with similar confluency and cryopreserved. If the well did not reach confluence within 3 weeks, the cells from that well were discarded. The remaining confluent wells were harvested and pooled by the end of the third week.

### REP culture

On day 0, 1 × 10^6^ TILs were thawed and cultured in a T-25 flask with 10 mL of REP culture medium. Next, 2 × 10^8^ irradiated feeder cells, 30 ng/mL OKT anti CD3 antibody, and 3000 IU/mL rhIL-2 were added to the flask. During the first week, once the cells in the flask reached confluence, half of the medium was replenished. On day 7, the cells were transferred to a T-75 flask with 40 mL of AIM-V medium. The cells in the flask were split into 2 flasks when the cell concentration reached over 1.5 × 10^6^ cells/mL. Cells were harvested by the end of the second week.

### REP culture setup using G-Rex 10M flask

On day 0, 1 × 10^6^ TILs were thawed and cultured in a G-Rex 10M flask with 40 mL of REP culture medium. Next, 2 × 10^8^ irradiated feeder cells, 30 ng/mL OKT anti CD3 antibody, and 3000 IU/mL rhIL-2 were added to the flask. On day 4, 3000 IU/mL IL-2 was added to 20 mL fresh REP culture medium, and the mixture was added to the flask. On day 7, 3000 IU/mL rhIL-2 was added to 40 mL fresh AIM-V medium and added to the flask. The cells in each flask were split into 2 flasks when the cell concentration reached over 3 × 10^6^ cells/mL. Cells were harvested by the end of the second week. Post-harvest beads removal was performed for two times by using DynaMag-50 Magnet (Thermo Fisher Scientific, Waltham, USA). Cell numbers were determined after beads removal.

### Antibodies for the detection of the phenotype and activity of TILs

The following fluorochrome-conjugated antibodies were used for flow cytometry: CD3-BV605 (Cat# 317322, BioLegend, San Diego, USA), CD4-eFluor506 (Cat# 69-0049041, Invitrogen, Waltham, USA), CD8-Pacific Blue (Cat# 300928, BioLegend, San Diego, USA), CD45-FITC (Cat# 368508, BioLegend, San Diego, USA), CD69-PE/Cyanine7 (Cat# 310911, BioLegend, San Diego, USA), CD103-BV711 (Cat# 350221, BioLegend, San Diego, USA), CD56-PE (Cat# 362507, BioLegend, San Diego, USA), CD14-PE (Cat# 301806, BioLegend, San Diego, USA), CD19-PE (Cat# 302208, BioLegend, San Diego, USA). 7-Amino-actinomycin D (7-AAD) staining solution (Cat# 420404, BioLegend, San Diego, USA) was used to identify nonviable cells. CD107a (Cat# 328639, BioLegend, San Diego, USA) was used as a marker of TIL degranulation.

### Cytotoxic T lymphocyte killing assay

On day 0, autologous tumor cells were thawed. Next, 1 × 10^5^ tumor cells per well were coated on a 96-well F-bottom cell culture plate. Autologous T cells were thawed and rested in a T-25 flask. On day 1, T cells were added to the plate that had been coated with autologous tumor cells at different effector-to-target (E:T) ratios. The supernatant of the coculture was harvested after 4 hours of incubation. Cells were collected from the supernatant, and their CD107a expression level was analyzed by flow cytometry to determine the cytotoxic degranulation activity of the expanded TILs.

Another plate of autologous tumor cells cocultured with TILs was prepared in the same way. In this case, the supernatant of the coculture was removed after 24 hours of incubation. Cells were then washed by adding 100 μL phosphate-buffered saline to each well. Fifty microliters of trypsin was added into each well to remove the cells from the plate. The viability of the tumor cells were analyzed by flow cytometry to determine the cytotoxic activity of autologous TILs. Tumor cells treated with 1% Triton X-100 were set as the positive control.

### Animal studies and tumor models

NOD.Cg-*Prkdc^scid^ Il2rg^tm1Wjl^*/SzJ (NSG) mice, 6 to 8 weeks of age and of both sexes, were purchased from The Jackson Laboratory (Bar Harbor, USA). The mouse care and handling procedures were approved by the Institutional Animal Care and Use Committee of The University of Texas MD Anderson Cancer Center.

To generate xenograft tumors, liposarcoma tumor fragments derived from patient YN020 were subcutaneously implanted into NSG mice. Tumors were measured with calipers twice weekly after implantation. Tumor volume was calculated by the formula V = (π/8) × (a b^2^), where V = tumor volume in cubic centimeters, a = maximum tumor diameter, and b = diameter at 90° to a. Mice received first injection of TILs or PBS when tumors reached 5mm in diameter. TILs (5×10^6^ cells/200 uL/mouse, n=5) or control PBS (200 uL/mouse, n=5) were i.v. injected into the mice for 2 times at intervals of 2 weeks. Mice were euthanized by CO_2_ inhalation when the tumor size exceeded 20 mm at the largest diameter in accordance with our approved protocol.

### H&E staining and Immunohistochemistry staining

Formalin-fixed paraffin-embedded sections were deparaffinized and stained with hematoxylin and eosin following the standard procedure.

For immunohistochemistry, formalin-fixed paraffin-embedded sections were deparaffinized and heated in antigen retrieval buffer. Tissue sections were first incubated with 3% H_2_O_2_ in distilled water for 20 min to block endogenous peroxidase activity and then in blocking buffer (5% normal horse serum and 1% normal goat serum in PBS) to block non-specific binding. The sections were incubated with primary antibodies, anti-CD3 epsilon antibody [SP7] (Cat# ab16669, Abcam Inc., Waltham, USA), overnight at 4°C and secondary antibody, Goat anti-Rabbit IgG (H+L) Secondary Antibody, HRP (Cat# A16096, Thermo Fisher Scientific, Waltham, USA), for 1 hour at room temperature. The nuclei were counterstained with hematoxylin. Slides were visualized under a Nikon Eclipse Ti fluorescence microscope.

### Statistical analysis

Data represented as scatter plots show individual patient data. Error bars represent standard error of the mean. Two-sided paired *t*-tests were used to compare samples under 2 different conditions for the *in vitro* study. Unpaired *t*-tests were used to compare tumor volume size from the TILs treated group vs. control group. To compare multiple groups under one condition, one-way ANOVA with Tukey multiple comparison test was performed. Analyses were conducted by using GraphPad Prism software. Significance was defined as *P* < 0.05.

## Results

### Liposarcoma and osteosarcoma tissue collection for TIL expansion

To evaluate the feasibility of our anti-CD137–independent and CD3/CD28 activator-based TIL expansion protocol, we collected 11 sarcoma specimens from patients who underwent surgical resection. Patients were selected without regard to age, sex, ethnicity, tumor location, and tumor type to maximize the tumor tissue heterogeneity for TIL expansion. The patients’ ages ranged from 18 to 72 years, with a median of 67. The ratio of dedifferentiated to well-differentiated for liposarcoma was 1:2. One-third of the specimens were collected from the retroperitoneum and the rest were collected from the scrotum, trunk, and other soft tissues. Four patients (YN024, YN025, YN028, and YN029) had not received any form of chemotherapy or radiotherapy before surgery, two patients (YN010 and YN030) had received both chemotherapy and radiotherapy, and the rest had received either chemotherapy or radiotherapy. In addition, we received one neuroblastoma sample from a patient who underwent surgical resection (YN033). We also tested our TIL expansion protocol on this non-sarcoma solid tumor. The clinical characteristics of the patients are summarized in (**Table 1**).

**Table 1:** Clinical information of 11 enrolled patients with sarcoma.

Patient number	Diagnosis	Progression status	Organ/site	Tissue type	Age (y)	Sex	Re-excision	Preoperative radiotherapy	Preoperative chemotherapy
YN010	Liposarcoma	Well-differentiated	Retroperitoneum	T	72	M	N	Y	Y
YN013	Liposarcoma	De-differentiated	Retroperitoneum	T/M T	58	M	N	N	Y
YN020	Liposarcoma	De-differentiated	Groin	T	47	M	N	Y	Y
YN023	Liposarcoma	De-differentiated	Retroperitoneum	T	53	F	N	Y	N
YN024	Liposarcoma	Well-differentiated	Trunk	T	66	M	N	N	N
YN025	Liposarcoma	Well-differentiated	Trunk	T	67	M	N	N	N
YN026	Spindle cell sarcoma	Lung metastasis	Lung	T	18	M	N/A	Y	N
YN027	Osteosarcoma	Tumor-metastasis	Chest wall	T	21	M	N/A	N	Y
YN028	Liposarcoma	Well-differentiated	Arm	T	40	M	N	N	N
YN029	Liposarcoma	Well-differentiated	Scrotum	T	69	M	N	N	N
YN030	Liposarcoma	Well-differentiated	Scrotum	T	68	M	N	Y	Y
YN033	Neuroblastoma	Metastasis	Left adrenal gland	T	5	M	N/A	N/A	N/A

T indicates tumor tissue; T/M indicates tumor margin tissue. Several patients who underwent resection had more than one tissue sample available. Tissue samples were numbered by the clinical collection team.

### Assessment of successful pre-REP TIL generation

To develop the anti-CD137–independent protocol for isolating and expanding TILs from liposarcoma and osteosarcoma, we reviewed the published protocol for the expansion of TILs for clinical use (11). Based on this review, we decided to use CTS Dynabeads CD3/CD28 to replace the anti-CD137 agonist antibody and to reduce the pre-REP time to 2 weeks in order to generate “young”—that is, minimally cultured—TILs.

**Figure 1:**
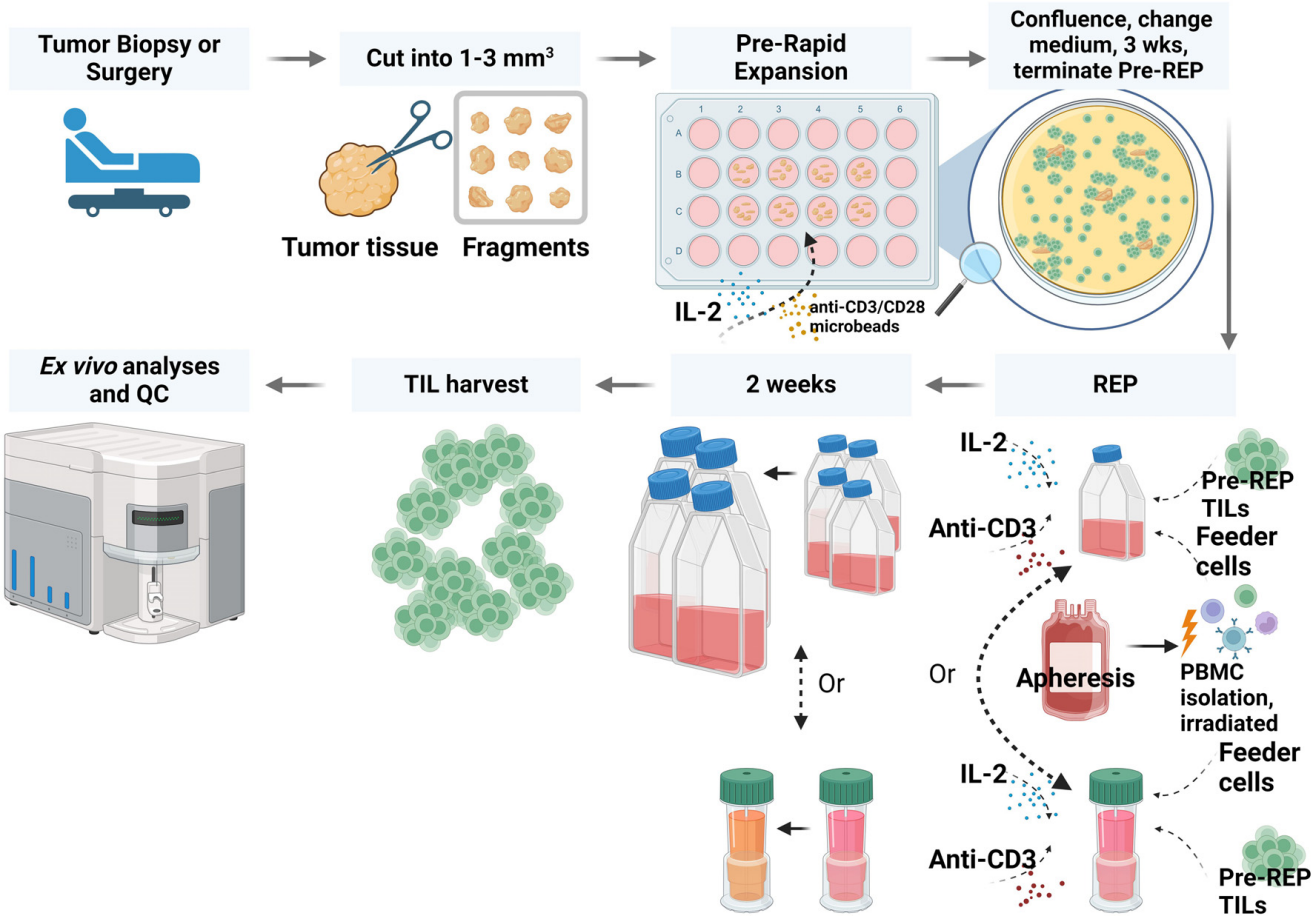
Workflow for preparing TILs from liposarcoma or osteosarcoma tissue. In brief, fresh tumor tissue specimens were collected and cut into fragments. TILs were expanded through a pre-rapid expansion phase (REP) and a REP. After TIL expansion and harvest, TIL phenotype and function were analyzed. QC, quality control; PBMC, peripheral blood mononuclear cell. The figure was created with BioRender.com.

Freshly resected specimens were processed, and pre-REP TILs were generated as described in the Methods section. Briefly, TILs were grown from the tumor fragments in high-dose IL-2 conditions and stimulated with anti-CD3/CD28 microbeads at day 0 as shown in (**Figure 1**). Pre-REP TILs were primed and expanded successfully from all 11 sarcoma patients’ samples. The expansion time for most pre-REP TILs (10/11) was between 12 and 17 days and yields of pre-REP TILs per tissue were between 1 × 10^6^ and 9 × 10^6^, as shown in (**Table 2**). A comparison of yield by patients’ treatment status found that previous treatment did not affect the yields of pre-REP TILs. We also found that age did not affect the TIL expansion ability. No significant difference of pre-REP TIL yields was observed between age groups (>40 years vs. <40 years, *P* = 0.097).

**Table 2:** Expansion of pre-rapid expansion phase (pre-REP) TILs from sarcoma tissue: duration and yields.

Patient Number	Conditions	Reactivity	Duration (days)	Yield
YN010	Fragments, aCD3/28, 6000 U/mL IL-2	Tissues 18, 19 +	28	Tissue 18 4.5 × 10^6^ Tissue 19 3.9 × 10^6^
YN013	Fragments, aCD3/28 6000 U/mL IL-2	Tissue1, 2 + Tissue 3 -	16	Tissue 1 8 × 10^6^ Tissue 2 8.5 × 10^6^
YN020	Fragments, aCD3/28 6000 U/mL IL-2	Tissue 5 + Tissue 6 +	16	Tissue 5 2.28 × 10^6^ Tissue 6 3.83 × 10^6^
YN023	Fragments, aCD3/28 6000 U/mL IL-2	Tissue 6 + (only 1 well showed activation)	14	Tissue 6 1.14 × 10^6^
YN024	Fragments, aCD3/28 6000 U/mL IL-2	Tissue 5+ Tissue 6+	12	Tissue 5 1.03 × 10^6^ Tissue 6 1.3 × 10^6^
YN025	Fragments, aCD3/28 6000 U/mL IL-2	Tissue 4+ Tissue 5+	13	Tissue 4 9 × 10^6^ Tissue 5 7.3 × 10^6^
YN026	Fragments, aCD3/28 6000 U/mL IL-2	Tissue 2 +	13	Tissue 2 8.95 × 10^6^
YN027	Fragments, aCD3/28 6000 U/mL IL-2	Tissue 2+	13	Tissue 2 5 × 10^6^
YN028	Fragments, aCD3/28 6000 U/mL IL-2	Tissue 4+ Tissue 5+	14	Tissue 4 3.12 × 10^6^ Tissue 5 1.08 × 10^6^
YN029	Fragments, aCD3/CD28 6000 U/mL IL-2	Tissue 4+ Tissue 5 +	17	Tissue 4 0.4 × 10^6^ Tissue 5 2.5 × 10^6^
YN033	Fragments, aCD3/CD28 6000 U/mL IL-2	Tissue 2+	21	Tissue 2 8.46 × 10^6^

+ indicates robust activation of T cells; – indicates no or minimal activation of T cells. All fragments were prepared at regular size (1-3 mm^3^) with antibiotic supplementation.

### Assessment of successful REP-TIL expansion

REP TILs were expanded following the classical protocol (11). In brief, in the absence of the anti-CD137 agonist antibody, pre-REP TILs were cultured with IL-2, OKT3 anti-CD3 antibody, and irradiated PBMCs as feeder cells. REP-TILs were obtained successfully from 10 of 11 patient samples. The minimum and maximum expansion ranged from 200- to 600-fold during a 14- to 15-day span (**Table 3**). Several advantages have been reported for the use of the gas-permeable G-Rex flask during the REP, including a static growth environment for TILs and promotion of consistent cell expansion (12). To shorten the expansion duration or the potential needs for additional TILs, we performed the REP TIL expansion in the G-Rex bioreactor. Given the same expansion time, compared with expansion in the T-75 flask, an additional 2- to 3-fold increase was achieved. The viability of REP TILs obtained from the G-Rex was slightly higher, and the quality of the T cells was about the same as those expanded in the T-75 flask. In summary, the addition of the G-Rex bioreactor to this anti-CD137 agonist antibody–independent protocol boosted the feasibility of the method for clinical application.

**Table 3:** Expansion of rapid expansion phase (REP) TILs from sarcoma tissue: duration and yields.

Patient Number	Conditions	Reactivity	Duration (days)	Yield
YN013	3000 U/mL IL-2 PBMC feeder cells 30 ng/mL OKT-aCD3 T-75	Tissues 1, 2 +	14	T-75 3.94 × 10^8^
	3000 U/mL IL-2 PBMC feeder cells 30 ng/mL OKT-aCD3 G-Rex	Tissue 2 +	14	G-Rex 9.96 × 10^8^
YN020	3000 U/mL IL-2 PBMC feeder cells 30 ng/mL OKT-aCD3 T-75	Tissue 6 +	15	T-75 4.8 × 10^8^
YN024	3000 U/mL IL-2 PBMC feeder cells 30 ng/mL OKT-aCD3 T-75	Tissue 5 +	15	T-75 6.4 × 10^8^
YN025	3000 U/mL IL-2 PBMC feeder cells 30 ng/mL OKT-aCD3 T-75	Tissue 4 +	14	T-75 3.68 × 10^8^
YN026	3000 U/mL IL-2 PBMC feeder cells 30 ng/mL OKT-aCD3 T-75	Tissue 2 +	15	T-75 <1 × 10^8^
YN027	3000 U/mL IL-2 PBMC feeder cells 30 ng/mL OKT-aCD3 T-75	Tissue 2 +	14	T75 1.5 × 10^8^
YN028	3000 U/mL IL-2 PBMC feeder cells 30 ng/mL OKT-aCD3 T75	Tissue 4 +	14	T75 2.2 × 10^8^
	3000 U/mL IL-2 PBMC feeder cells 30 ng/mL OKT-aCD3 G-Rex	Tissue 4 +	14	G-Rex 4.88 × 10^8^
YN029	3000 U/mL IL-2 PBMC feeder cells 30 ng/mL OKT-aCD3 T-75	Tissue 5 +	14	T75 2.4 × 10^8^
YN033	3000 U/mL IL-2 PBMC feeder cells 30 ng/mL OKT-aCD3 T-75	Tissue 2 +	14	T75 1.8 × 10^8^

+ indicates robust TIL expansion at the end of the REP. All studies were performed using T-75 flasks for culture. The G-Rex protocol for generation of REP TILs was tested in samples YN013 and YN028. The same sources of pre-REP TILs for seeding culture were used as for the T-75 flask protocol.

### Phenotypic analysis of pre-REP and REP TILs

**Figure 2:**
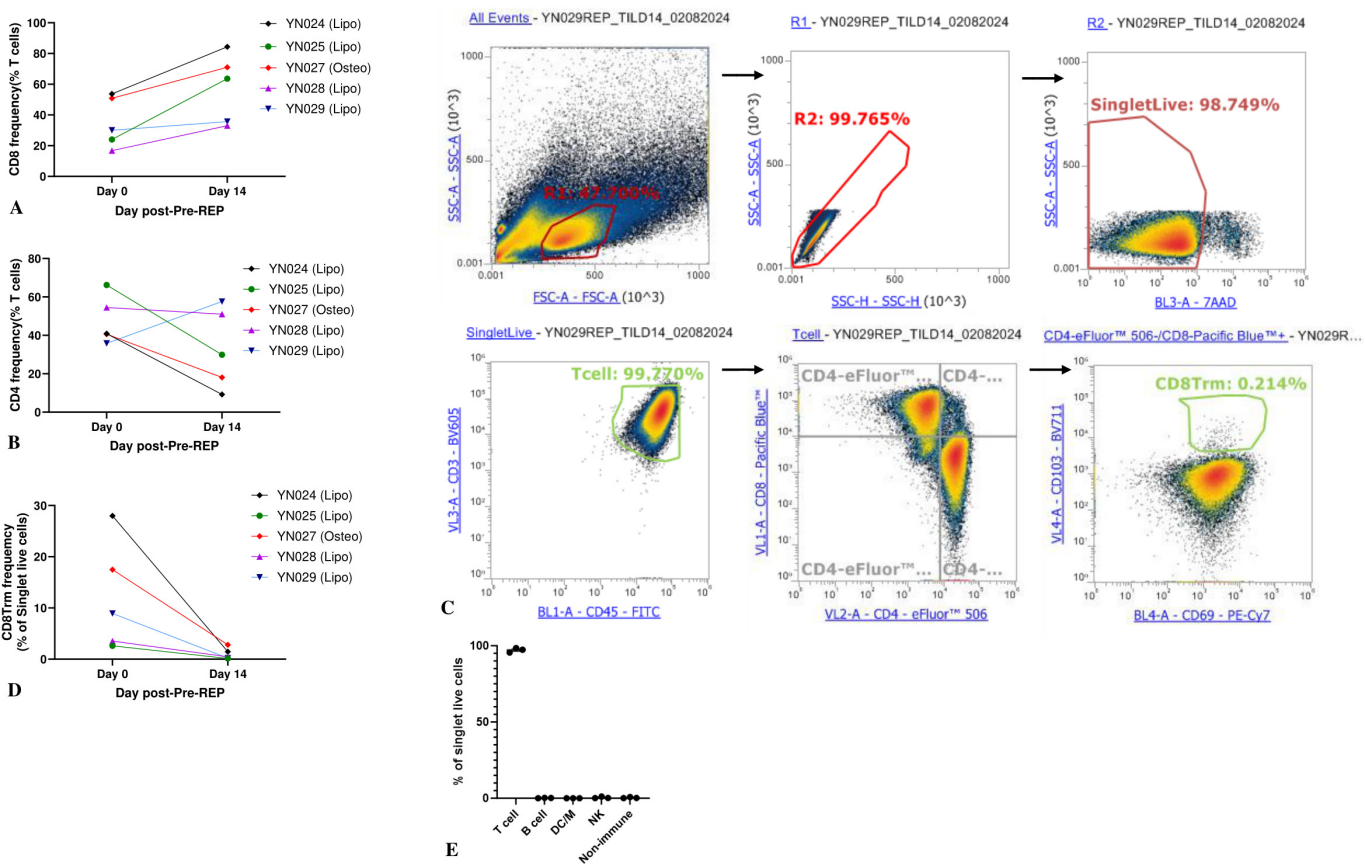
Characterization of TIL phenotypes at the end of pre-REP (day 0) and REP (day 14). **(A)** Proportion of CD8+ T cells among TILs harvested at pre-REP vs. REP. Two-sided paired *t*-test, *P* < 0.05; *N* = 5. **(B)** Proportion of CD4+ T cells among TILs harvested at pre-REP vs. REP. Two-sided paired *t*-test; *N* = 5. **(C)** Gating strategy for CD8+ T_RM_ cells (CD3+CD8+CD69+CD103+). **(D)** Proportion of CD8+ T_RM_ cells harvested at pre-REP vs. REP. Two-sided paired *t*-test; *N* = 5. **(E)** Composition of TIL culture after REP. Frequencies of T cells (CD3+CD45+), B cells (CD3−CD19+), dendritic cells/macrophages (CD3−CD14+), and NK cells (CD3−CD56+) were measured. *N* = 3. DC/M, dendritic cells/macrophages; NK, natural killer.

Different expansion protocols, methods, and initial tumor types can generate TIL populations with different compositions (13). In general, a high CD8:CD4 ratio is favored because it is a good indicator of effective TIL therapy and short expansion time (14). We performed flow cytometric analysis of the phenotypes of the pre-REP TILs and REP TILs at the end of each expansion phase. Three samples of pre-REP TILs were predominantly composed of CD4+ T cells, while in 2 samples (YN024 and YN027), over 50% of the T cells were CD8+ T cells. However, the REP phase skewed the phenotype towards CD8+ T cells from 35% ± 7% at the end of pre-REP to 57.5% ± 10% at the end of the REP, as shown in (**Figure 2A**). On the contrary, CD4+ T cells were suppressed in most samples, except sample YN029, as shown in (**Figure 2B**).

Tissue-resident memory T cells (T_RM_) are a heterogeneous T-cell population that resides in the tissue and plays a long-term immune-protective role. Recent studies have suggested that T_RM_ cell infiltration correlates with enhanced response to currently available immunotherapies and is often associated with favorable prognosis (15). We measured the percentage of T_RM_ cells (CD3+CD4+CD8+CD69+CD103+) of all 5 samples (YN024, YN025, YN027, YN028 and YN029). The gating strategy for T_RM_ cells is displayed in (**Figure 2C**). Following a 2-week pre-REP expansion, TILs from all 5 samples displayed a mean frequency of CD8+ T_RM_ cells of 12% ± 4%. After a 2-week REP, the fraction of CD8+ T_RM_ cells was lower (1% ± 0.5%), though not significantly so (*P* = 0.06), as shown in (**Figure 2D**). The same pattern was observed in the CD4+ T_RM_ cells. Thus, our data demonstrated that our TIL products contain a fraction of T_RM_ cells and that a short *ex vivo* expansion time preserves that property.

TILs in the solid tumor microenvironment are heterogeneous and include the most abundant T cells and, less frequently, B cells, natural killer (NK) cells, dendritic cells, macrophages, and other types of cells. Non-T cells could also come out of the tumor fragments into the culture medium when the growth of T cells was initiated. Although the high-dose IL-2 and anti-CD3/CD28 Dynabeads method creates an environment favorable for T-cell growth, it has been reported that a high level of IL-2 can evoke the proliferation and activation of NK cells (16). Therefore, it is critical to assess the composition of the TIL culture after the REP for quality-control purposes. We analyzed the phenotypes of the 5 patient-derived pre-REP TIL cultures by flow cytometry. Analysis of REP TILs showed that the majority of the cells were CD3+ T cells (mean: 88% ± 5%, range: 79%-96%), whereas only 4% ± 3% were CD56+CD3− NK cells. The percentages of T cells (CD3+CD45+), B cells (CD3−CD19+), dendritic cells/macrophages (CD3−CD14+), NK cells (CD3−CD56+), and other nonimmune cells (CD45−) are shown in (**Figure 2E**).

### Expanded TILs kill PDX-derived autologous tumor target cells

**Figure 3:**
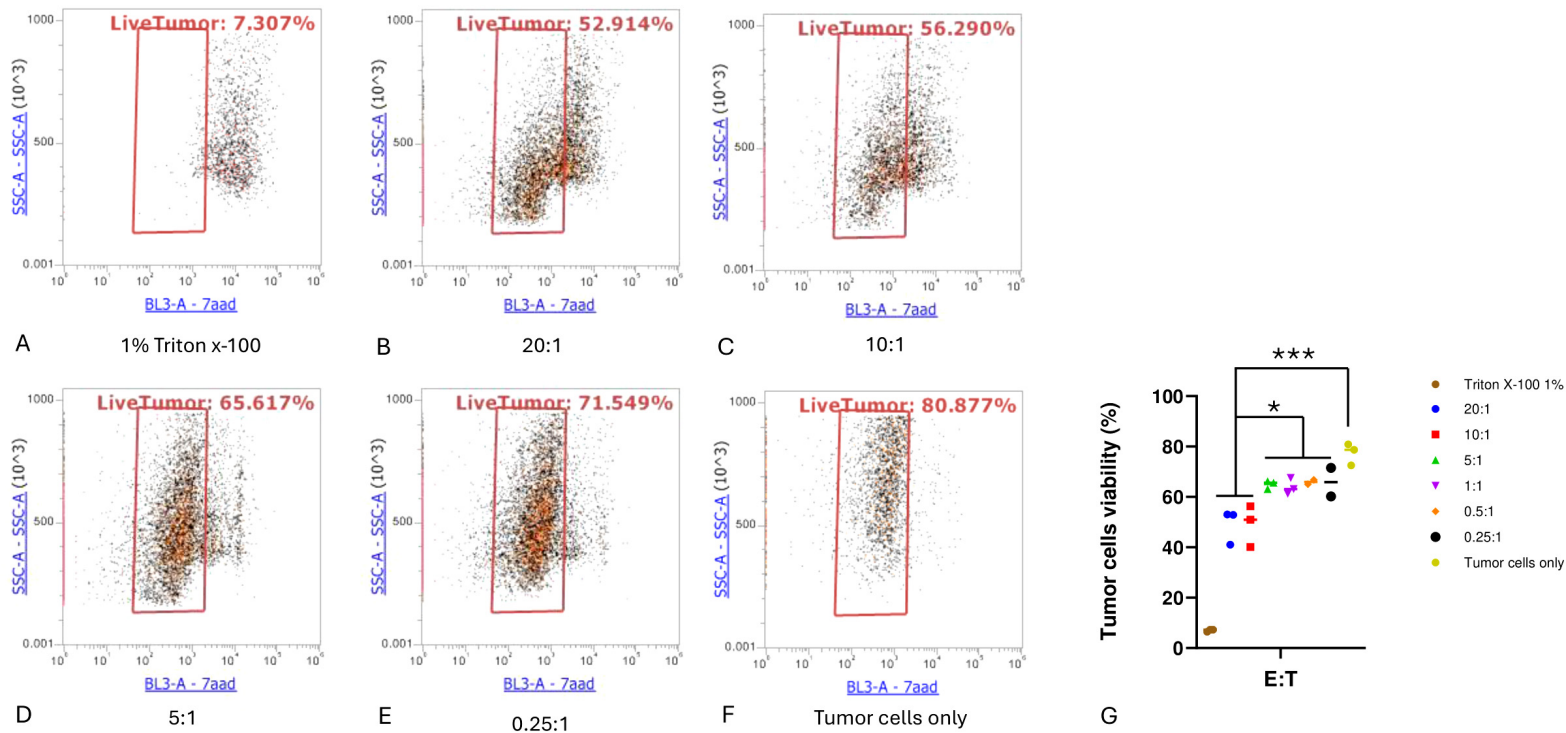
Cytotoxic T lymphocyte tumor cell killing assay. **(A)** Positive control well (1% Triton X-100); **(B–E)** indicated effector-to-target cell (E:T) ratios; **(F)** tumor cells without T-cell treatment; **(G)** comparison of viable cells for each condition. Experiments were performed in triplicate for each E:T ratio. One-way ANOVA with Tukey multiple comparison analysis; *P < 0.05; ***P < 0.001.

To verify the biological function and tumor specificity of the TILs generated from our protocol, we derived autologous tumor cells from PDX tumors and measured cytotoxic T lymphocyte (CTL) activity. T cell–mediated killing was quantified by measuring the viability of the remaining tumor cells after coculture. In brief, 24 hours after coculture, the suspension of TILs was removed. Attached cells were washed, detached, stained, and analyzed by flow cytometry. The gating of the liposarcoma tumor cells was first determined by analyzing the sample containing only the tumor cells. Notably, 99% of the sample containing only tumor cells were CD45−. On the contrary, the sample containing tumor cells cocultured with TILs contained a small portion of CD45+ cells because of the residual T cells. Therefore, CD45− was used for gating liposarcoma tumor cells. After treatment with TILs at an E:T ratio of 20:1, 52% of live tumor cells remained, a significant reduction (*P* < 0.001) compared to the tumor cells cultured alone (80% viability), as shown in (**Figures 3B, F**). Similar results were observed at an E:T ratio of 10:1, as displayed in (**Figure 3C**). Tumor cells treated with 1% Triton X-100 were set as the positive control (9% viability), as shown in (**Figure 3A**).

### Activation and cytotoxic degranulation activity of expanded TILs after coculture with autologous tumor cells

**Figure 4:**
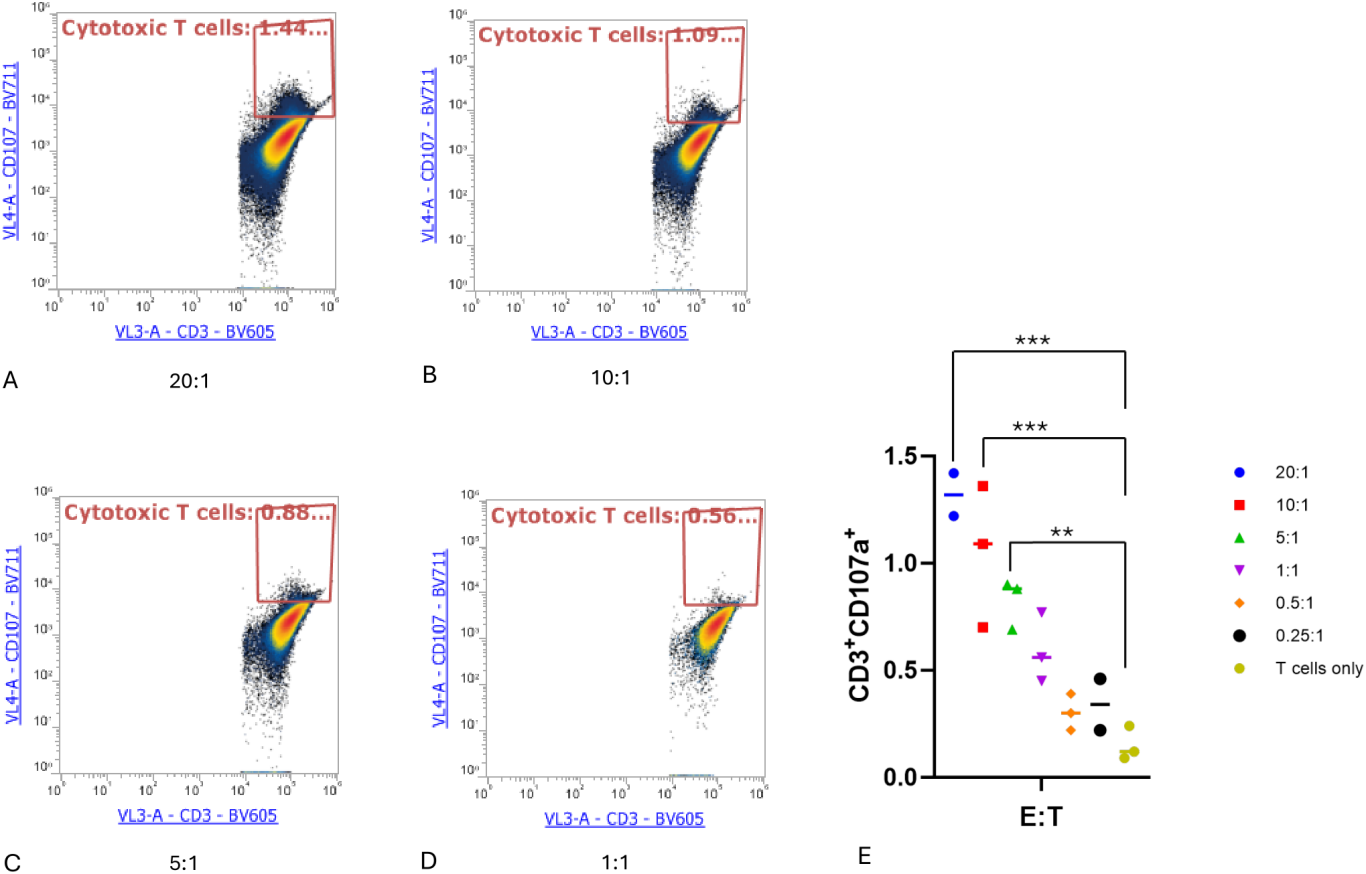
Cytotoxic T lymphocyte CD107a degranulation assay. Cytotoxic T lymphocytes (CTLs) were defined as CD3+CD107a+. **(A–D)** CTLs at the indicated effector-to-target cell (E:T) ratios. **(E)** Proportion of cytotoxic T cells in each condition. One-way ANOVA with Tukey multiple comparison analysis; **P < 0.01; ***P < 0.001. Triplicates of experiments were performed for each condition.

Tumor cells can be recognized and killed by activated cytotoxic CD8+ T cells through the secretion of lytic granules that kill target cells (17). When this lytic function is activated, degranulation occurs on CD8+ T cells, upon which CD107a is exported to the T-cell surface (18). Therefore, we measured the surface marker CD107a to determine the activation and degranulation activity of expanded TILs. TILs were treated as effector cells and cocultured with target autologous tumor cells at different E:T ratios for 4 hours. Cells in the suspension were then harvested, stained with antibodies, and analyzed by flow cytometry. In accordance with the cytotoxicity assay results, both the TILs cocultured with tumor cells at an E:T ratio of 20:1 and those cultured at an E:T ratio of 10:1 exhibited significantly higher levels of CD107a compared to cultures containing only T cells (*P* < 0.001). In addition, the T cells cocultured at an E:T ratio of 5:1 had an increased level of CD107a compared to T cells alone (*P* < 0.05), as shown in (**Figure 4**).

### Transfer of autologous TILs reduced tumor size

**Figure 5:**
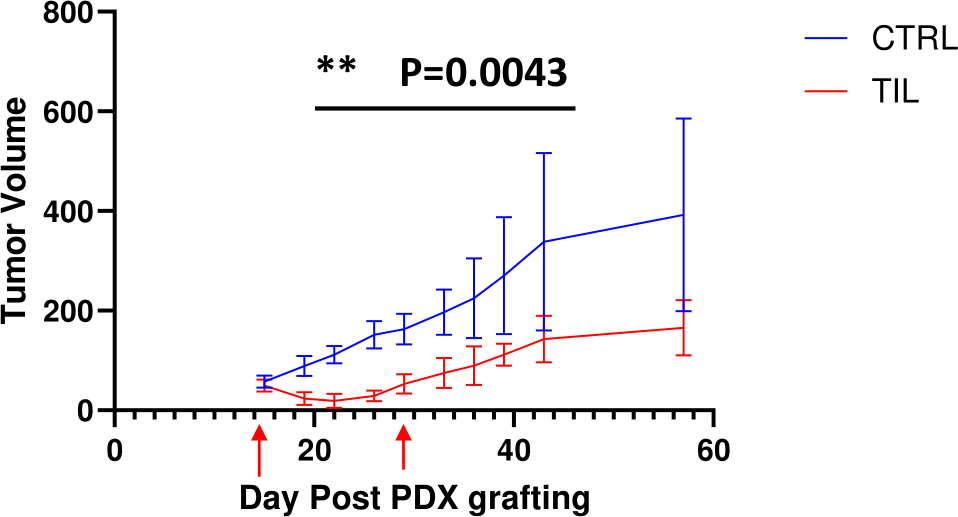
Transfer of TILs showed significant antitumor efficacy against human PDX tumors. Liposarcoma tumors were embedded subcutaneously in NSG mice (n=5/group). Tumor-bearing mice were preconditioned when tumors reached 5 mm in diameter, followed by T-cell injection (red arrows) or PBS. Tumors were measured twice per week. The results represent two repeated experiments. The graphs showed the tumor size (± SEM) at each timepoint of measurement. Unpaired *t*-tests; **P < 0.01.

Inspired by the result that expanded TILs kill PDX-derived autologous tumor target cells *in vitro*, we next explored the potential antitumor efficacy of TILs against solid tumors *in vivo*. Because PDX tumor models retain the original tumor architecture from patients as well as the genetic profile, we developed osteosarcoma PDX model (YN020) and tested the effectiveness of the autologous TILs treatment in these osteosarcoma bearing mice. Mice were subcutaneously implanted with patients’ derived tumor fragments and started to receive treatment when tumors reached 5 mm in diameter. Compared with the rapid tumor growth in the PBS-treated control group, tumor development was remarkably delayed and stabilized by autologous TILs in the TILs injection group, as shown in (**Figure 5**) (*P* < 0.05). The tumor size difference between two groups can be visualized in (**Supplementary Figure S1**). To study TIL infiltration in tissues, we stained section of tumors from PDX-bearing mice at the end of the study (**Supplementary Figures S2**, **S3**). TILs were detected both inside and at the edge of the tumors of PDX-bearing mice (**Supplementary Figures S3A, B**) but not in control group mice (**Supplementary Figures S3C, D**). These results indicated that TIL treatment infiltrated into the tumors and effectively restricted tumor growth on PDX-bearing mice.

## Discussion

Despite the established, standard treatment regimens of surgical removal, radiotherapy, and chemotherapy for patients with sarcoma, major concerns remain, including the heterogeneity of response to therapy and the high recurrence and metastasis rates (19–23). Insights into the genomic landscape, immunological and molecular features of the sarcoma, and interactions between immune cells and tumor microenvironment have advanced therapies in many types of soft tissue sarcomas (24–26). Therapeutic strategies focused on genetic mutations or specific molecules displayed promising efficacy in several sarcomas and fewer side effects were reported compared to traditional therapies (24, 25). However, drug resistance, limited duration of efficacy and no specific mutations in some sarcomas limited the development of the target therapy (25). These challenges required to adopt the therapy strategies with a relative long-term effect but regardless of drug resistance and mutation markers of sarcomas. This led to the application of immunotherapy such as immune checkpoint inhibitors (ICIs) against sarcoma (26). Multiple ICIs that target the PD-1/PD-L1 pathway have been tested in patients with sarcoma, but the only approval is in a rare subtype of alveolar soft-part sarcoma. Encouraging activity has been reported with ICIs in patients with undifferentiated pleomorphic sarcoma or dedifferentiated liposarcoma (23, 27). However, given that a wide variety of sarcoma subtypes with low objective response rates, the overall efficacy of ICIs is not entirely satisfactory in sarcomas. Indeed, similar outcomes have been observed with ICI treatment of other types of cancer; there is always a fraction of tumors that are refractory to these treatments (28). Thus, ICI treatment requires selection of optimal candidates and a more immunogenic tumor. In contrast, ACT with endogenous TILs displays promising clinical benefits for patients with several types of solid tumors. In the past decades, clinical trials have demonstrated good responses to TIL therapy, mainly in melanoma, cervical cancer, breast cancer, and non-small cell lung cancer (29). An ongoing clinical trial (NCT03935893) is studying adoptive transfer of TILs for advanced solid tumors including sarcoma, and another (NCT04052334) is evaluating the safety and feasibility of TIL treatment with high-dose IL-2 in patients with soft tissue sarcoma (30, 31). Surprisingly, however, a GMP protocol for isolation and expansion of TILs from sarcoma tumors is not available. This is probably because (1) TIL treatments for immunologically nonresponsive “cold” tumors have not been explored extensively (2); the heterogeneity of sarcoma makes it a challenged target for which to design and produce TILs; and (3) the discontinued supply of the anti-CD137 agonist antibody halted the process of generating selective antitumor TILs and could be detrimental to the clinical efficacy of TIL-based ACT. Therefore, we developed a TIL preparation method that is clinically feasible under GMP conditions.

The classical approach for producing TILs usually begins with a pre-REP expansion phase. TILs are first either isolated from a single-cell suspension after the enzymatic digestion of the tumor tissue or directly cultured from small fragments of fresh tumor tissue followed by *in vitro* expansion with a high dose of IL-2. We chose to start TIL culture from small tumor fragments for 2 reasons: first, contamination of resected primary tumors is detrimental but can be monitored during pre-REP. By regularly checking the initial culture for contamination and growing the TILs in separate wells, we decreased the risk of contamination and were able to exclude wells with contamination at an early stage. Second, competition between CD8+ T cells and CD4+ T cells has been reported in the TIL expansion procedure (32, 33). With the digestion method followed by IL-2 expansion, the generated TILs could lose their TCR repertoire diversity and only present the dominant clone types. However, inducing TILs from fragments in individual wells could maximally preserve the TCR repertoire diversity, assuming that adequate numbers of T cells can be obtained in each well or from 1 or 2 pooled wells at the end of pre-REP. Considering the potential downstream applications for discovering the tumor-reactive TCR, our method provides a feasible solution to the problem of TCR diversity loss.

The tumor necrosis factor CD137 provides a co-stimulatory signal in activated T cells and enhances antitumor response (34). Several strategies targeting CD137 have been reported in the generation of TILs. One strategy is to add an anti-CD137 agonist antibody during the process. Preclinical studies have demonstrated that both the yield and antitumor activity of TILs increased after addition of the agonist antibody (34, 35). However, most of these preclinical studies used the research-grade antibody or received the GMP-grade CD137 antibody as a gift from the pharmaceutical company. There is no available GMP source of the antibody in the market. Another strategy is to enrich CD137+ tumor-reactive T cells. In this strategy, TILs are first sorted by their expression of CD137, and these enriched TILs are then selected by their reactivity to the autologous tumor cells (36, 37). In our protocol, instead of focusing on CD137, we adopted the “young TIL” strategy (38). The pre-REP bulk unselected TILs were generated within 2 to 3 weeks. Two reasons prompted us to choose this method: first, several studies have reported that TILs generated by the “young TIL” method showed similar clinical effects to those generated by the “selected TIL” method; second, we were able to avoid the challenges of generating autologous tumor cells and therefore save time in the selection of tumor-reactive TILs (39–41). To replace the anti-CD137 agonist, we added anti-human CD3/CD28 antibodies to stimulate the TIL expansion. This stimulation method has been proved to successfully elicit the expansion of TILs from several other types of solid tumor tissue (42, 43).

Cultured pre-REP TILs were propagated through the REP procedure with the help of irradiated PBMC feeder cells, IL-2, and OKT-anti CD3 antibody. After 2 weeks of incubation, REP TILs increased by 200- to 600-fold and CD8+ T cells were preferentially expanded. To test the antitumor efficacy of REP TILs *in vivo*, a PDX mouse model was generated by subcutaneously transplanting human-derived sarcoma tissue. Injection of PDX-bearing mice with autologous REP-TILs significantly shrank the tumor size compared with vehicle-injection controls. Taken together, we developed a clinically feasible protocol for expanding TILs from sarcoma tumor tissue and characterized the phenotype of these TILs. Both *in vivo* and *in vitro* assays confirmed that our TILs are reactive to autologous tumor cells.

It is worth noting that, although ACT with TILs provides unique advantages for treating solid tumors, a few limitations still constrain its application and clinical development. Ongoing research and the development of next-generation TILs have focused on the effort to augment both the numbers and activity of TILs to treat non-melanoma “cold” tumors. A significant challenge to applying TIL-based ACT to “cold” tumors is the lack of infiltrating T cells in the tumor tissue with which to start expansion. This lack of T cells could be attributed to several factors such as lack of tumor antigens, suppression of antigen presentation, or immunosuppression in the tumor environment (44, 45). To enrich for T cell tumor infiltration, combinations of ICI or radiotherapy with ACT can be used to release more antigens. Additionally, researchers have sought to identify neoantigens to activate and prime antigen-presenting cells (45, 46). To enhance the antitumor activity of TILs, engineered TILs that express IL-12, IL-15, or IL-21 have been tested (47, 48). To facilitate TIL homing and tumor infiltration, Forget et al. (49) expanded TIL from metastatic melanoma tumors and developed CXCR2-transduced TILs. Furthermore, for treating osteosarcoma, Yang et al. (50) developed membrane-anchored, tumor-targeted IL-12 (attIL12)-armed PBMCs, which can impede osteosarcoma growth. Building on this success, Hu et al. (51, 52) reported that attIL12-armed T cells can disrupt the extracellular matrix in osteosarcoma tumors, prompt T-cell infiltration, stimulate dendritic cell maturation, and induce antigen spreading. This novel approach and promising data lead us to consider attIL12 engineered-TIL trials as our next step to validate our protocol clinically. Given that multiple mechanisms underlie ACT resistance, it is unlikely that a single strategy for ACT with TILs can treat all solid tumors. We expect that combining different treatments into tailored strategies for different types of solid tumors will overcome these challenges in the future.

Limitation of the study: We recognize several limitations of our current work. First, the exhaustion status of TILs product was not characterized. Exhausted TILs will impair their anti-tumor efficacy and limit their response to other immunotherapy treatments such as ICI if a combination of treatment strategies is considered. Therefore, inhibitory markers such as PD-1, LAG-3, and TIM-3 from TILs will be determined in future studies. Second, we have not compared the TIL’s expansion yield, anti-tumor efficacy, clones’ diversities, exhaustion status by using T cell activator CD3/CD28 beads vs. anti-CD137 agonist antibody protocol. While important, this was beyond the scope of the current study which was dedicated to developing a GMP-adherent method for expanding endogenous TILs without anti-CD137 agonist. Future studies to compare the characteristics and functional capabilities of the generated TILs under the research settings by using the current protocol vs. using research grade anti-CD137 agonist are warranted.

## Data Availability

The original contributions presented in the study are included in the article/**Supplementary Material**. Further inquiries can be directed to the corresponding authors.

## Supplementary material

Supplementary Figure 1 TIL treatment restrained the growth of PDX tumors. The images of tumors corresponding to the tumor growth in **Figure 5** and were taken post-PDX graft at day 57. **(A)** tumor from the mice received TIL treatment. **(B)** tumor from the control group mice.

Supplementary Figure 2 H&E staining of tumor tissues. Tumor tissues were collected at the end of the study, fixed in 10% formalin and subjected to H&E staining. Scale of 100 µm. **(A)** tumor tissue from a control mouse. **(B)** tumor tissue from a mouse received TIL treatment.

Supplementary Figure 3 IHC staining of immune cells of tumor tissues. T-cell infiltration in tumors from PDX tumor-bearing mice were determined by anti-human CD3 immunohistochemistry staining at the end of the study. Scale of 20 µm. **(A)** Presence of CD3 T cells inside of the tumor from mice received TIL treatment. **(B)** Presence of CD3 T cells at the edge of tumor from mice received TIL treatment. **(C)** No presence of CD3 T cells inside of the tumor from control group mice. **(D)** No presence of CD3 T cells at the edge of tumor from control group mice.
