# Supplementary figures and images for: Anti-CD137 agonist antibody–independent and clinically feasible preparation of tumor-infiltrating lymphocytes from soft tissue sarcoma and osteosarcoma
Source: Front Immunol. 2025 Mar 12;16:1557006. doi: 10.3389/fimmu.2025.1557006 (PMC11936977; doi:10.3389/fimmu.2025.1557006)

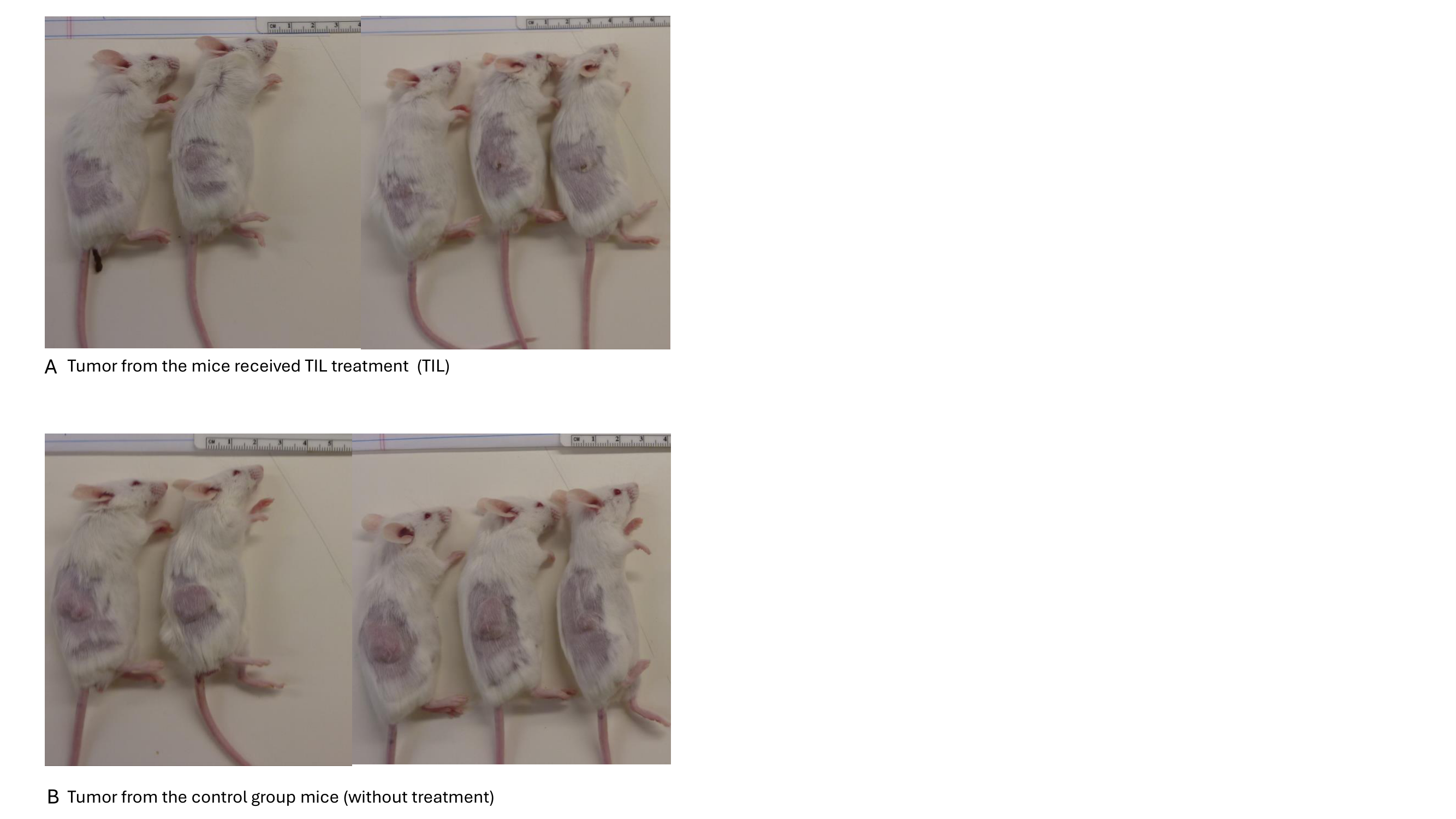

Supplement: Supplementary Figure 1 — TIL treatment restrained the growth of PDX tumors. The images of tumors corresponding to the tumor growth in Figure 5 and were taken post-PDX graft at day 57. (A) tumor from the mice received TIL treatment. (B) tumor from the control group mice. [file Image1.tiff]

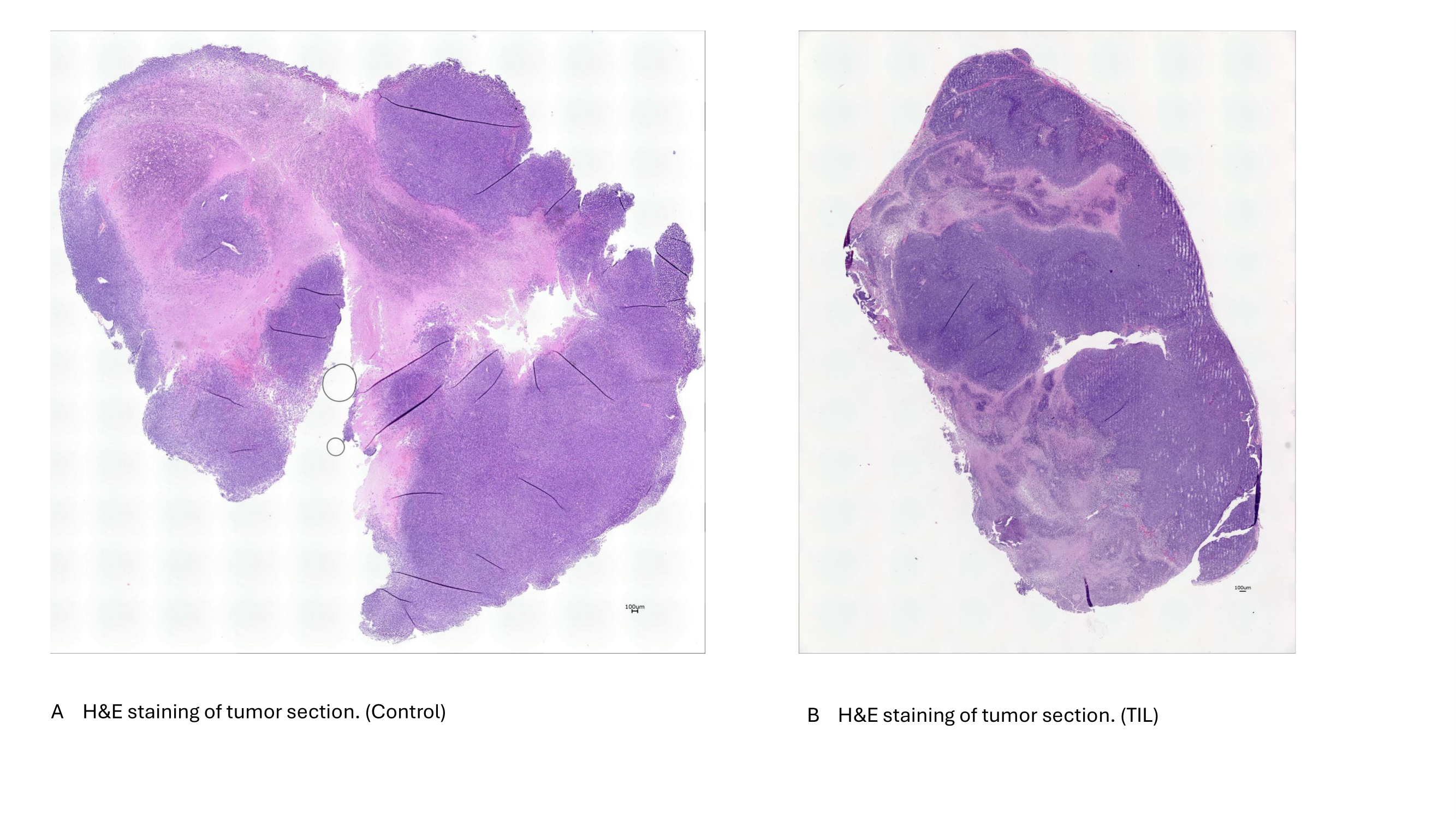

Supplement: Supplementary Figure 2 — H&E staining of tumor tissues. Tumor tissues were collected at the end of the study, fixed in 10% formalin and subjected to H&E staining. Scale of 100 µm. (A) tumor tissue from a control mouse. (B) tumor tissue from a mouse received TIL treatment. [file Image2.tiff]

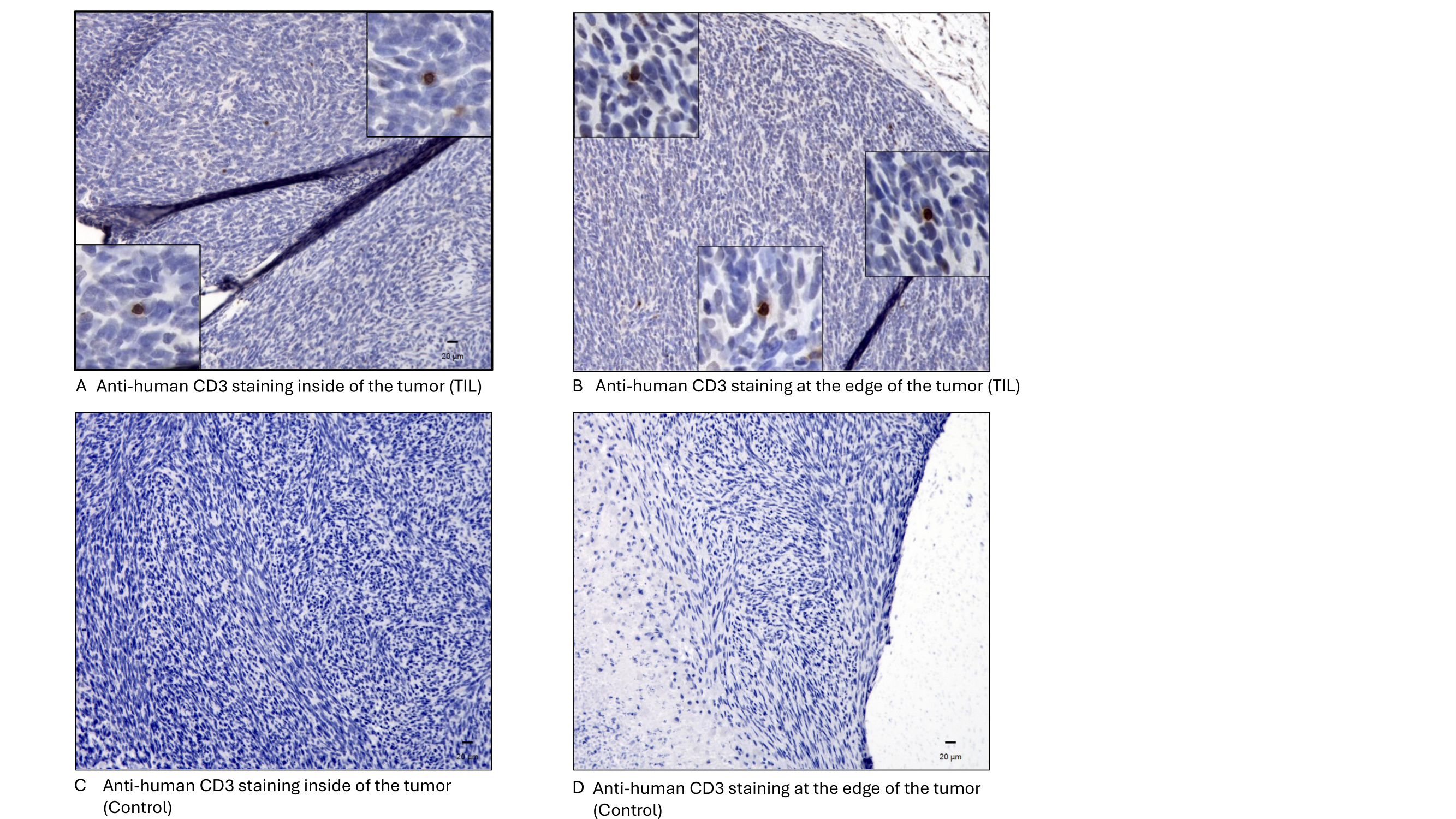

Supplement: Supplementary Figure 3 — IHC staining of immune cells of tumor tissues. T-cell infiltration in tumors from PDX tumor-bearing mice were determined by anti-human CD3 immunohistochemistry staining at the end of the study. Scale of 20 µm. (A) Presence of CD3 T cells inside of the tumor from mice received TIL treatment. (B) Presence of CD3 T cells at the edge of tumor from mice received TIL treatment. (C) No presence of CD3 T cells inside of the tumor from control group mice. (D) No presence of CD3 T cells at the edge of tumor from control group mice. [file Image3.tiff]
